# Extrapolation-Based References Improve Motion and Eddy-Current Correction of High B-Value DWI Data: Application in Parkinson’s Disease Dementia

**DOI:** 10.1371/journal.pone.0141825

**Published:** 2015-11-03

**Authors:** Markus Nilsson, Filip Szczepankiewicz, Danielle van Westen, Oskar Hansson

**Affiliations:** 1 Lund University Bioimaging Center, Lund University, Lund, Sweden; 2 Department of Medical Radiation Physics, Lund University, Lund, Sweden; 3 Department of Radiology, Lund University, Lund, Sweden; 4 Imaging and Physiology, Skåne University Hospital, Lund, Sweden; 5 Clinical Memory Research Unit, Department of Clinical Sciences, Malmö, Lund University, Lund, Sweden; 6 Memory Clinic, Skåne University Hospital, Malmö, Sweden; Brighton and Sussex Medical School, UNITED KINGDOM

## Abstract

**Purpose:**

Conventional motion and eddy-current correction, where each diffusion-weighted volume is registered to a non diffusion-weighted reference, suffers from poor accuracy for high b-value data. An alternative approach is to extrapolate reference volumes from low b-value data. We aim to compare the performance of conventional and extrapolation-based correction of diffusional kurtosis imaging (DKI) data, and to demonstrate the impact of the correction approach on group comparison studies.

**Methods:**

DKI was performed in patients with Parkinson’s disease dementia (PDD), and healthy age-matched controls, using b-values of up to 2750 s/mm^2^. The accuracy of conventional and extrapolation-based correction methods was investigated. Parameters from DTI and DKI were compared between patients and controls in the cingulum and the anterior thalamic projection tract.

**Results:**

Conventional correction resulted in systematic registration errors for high b-value data. The extrapolation-based methods did not exhibit such errors, yielding more accurate tractography and up to 50% lower standard deviation in DKI metrics. Statistically significant differences were found between patients and controls when using the extrapolation-based motion correction that were not detected when using the conventional method.

**Conclusion:**

We recommend that conventional motion and eddy-current correction should be abandoned for high b-value data in favour of more accurate methods using extrapolation-based references.

## Introduction

Diffusion tensor imaging (DTI) and diffusion tensor tractography are widely used neuroimaging techniques that yield information on the tissue microstructure [1,2]. These techniques are based on the analysis of data acquired with relatively low diffusion weighting using a b-value of approximately 1000 s/mm^2^. Acquiring data using higher b-values, i.e., with *b* above 2000 s/mm^2^ enables the use of diffusional kurtosis imaging (DKI) [3], which is a technique that complements DTI and is sensitive to the within-voxel variability of diffusion tensors [4]. High angular resolution diffusion imaging (HARDI) for improved tractography in regions of crossing fibres also benefits from the use of high b-values [5,6]. Moreover, high b-value acquisitions serves as the basis for microstructure imaging, where the data is analysed using biophysical models in order to estimate physiologically important parameters such as the axon diameter or the cell-membrane permeability [7]. From a clinical perspective, high b-value data may enable, for example, improved tumour grading and better characterization of stroke lesions [8,9].

Prior to analysis of high b-value data, it must be corrected for subject motion and eddy-current distortions induced by the diffusion encoding gradients [10,11]. A simple approach to this problem is to register each diffusion-weighted volume to a reference volume acquired without diffusion encoding (b = 0 s/mm^2^). To account for both motion and eddy-current induced distortions, a full affine transform can be used with 12 degrees of freedom representing translation, rotation, shearing, and scaling along or around the x, y, and z axes [12]. Approaches other than the full affine transform can be used to reduce the complexity of the transform [13], or to fully model the distortion [11,14]. Regardless of the technique in use, the transform parameters are optimised using an objective function, which is commonly based on mutual information [15]. The choice of mutual information over a simpler metric, such as the sum-of-squares of the image difference, is motivated by the markedly different contrast in images acquired with and without diffusion encoding.

**Fig 1:**
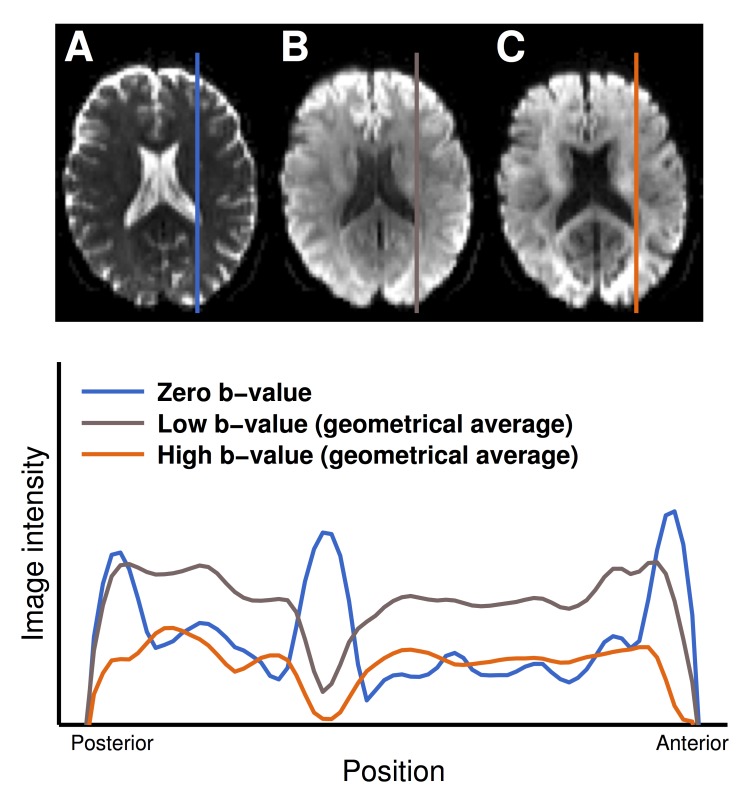
The challenge: Motion and eddy-current correction of diffusion MRI data in the elderly brain. Top row: Non diffusion-weighted MR-image (left), and diffusion-weighted images, encoded using a b-value of 1000 s/mm^2^ (middle) and 2750 s/mm^2^ (right) and averaged across multiple directions. Note that the rim of CSF surrounding the anterior part of the brain visualized in the non-diffusion weighted image to the left is absent in the diffusion weighted images. Bottom: Normalized plot of the logarithm of the MR signal as a function of position along the lines indicated in the images at the top. The anterior position where the MR signal turns into background is shifted posteriorly when comparing the zero b-value profile (blue) to the high b-value profile (orange). If the correction employs a non diffusion-encoded reference, the difference in contrast may cause the high b-value data to be erroneously scaled in the antero-posterior direction to fit the low b-value signal outline.

We will refer to the method where a full affine transform is used to register diffusion-weighted volumes to a non diffusion-weighted reference by optimizing the mutual information as the ‘conventional method’. Although mutual information metrics are designed to allow registration of images with different contrasts, the conventional method suffers from poor accuracy [16], mainly due to the large difference in contrast between low and high b-value images. This difference is most pronounced where a rim of CSF surrounds the brain, which is common in elderly subjects and patients with cerebral atrophy. This rim of CSF is clearly visible in non diffusion-weighted images, but is completely attenuated in high b-value images, which results in an inward shift of the apparent brain outline (Fig 1). This shift may induce errors in conventional motion and eddy-current correction of high b-value data, since image registration algorithms tend to match borders. Extrapolation-based correction, where reference volumes with appropriate high b-value contrast are extrapolated from low b-value data, was suggested by Ben-Amitay et al as a potential solution [16]. The benefit of such a method is that low b-value data can be corrected using the conventional method with a precision sufficient for accurate extrapolation of undistorted reference volumes. Ben-Amitay et al used standard DTI analysis on the low b-value data, and adapted the results for the CHARMED model in order to extrapolate to high b-value shells. However, standard DTI performs poorly in regions where tissue is mixed with free water such as CSF [17], which may have a negative impact on the extrapolation.

In this study, we compared conventional and extrapolation-based motion and eddy-current correction methods in a group of patients with Parkinson’s disease dementia (PDD) and healthy controls. We employed DTI as the starting point for extrapolations, and compared the CHARMED-based extrapolation with a novel method that employs partial-volume correction for CSF. Comparisons comprised the magnitude of registration errors between low and high b-value data, and we demonstrated the impact of the choice of motion and eddy-current correction method on the accuracy of tractography. Finally, we tested whether the choice of correction method can have an impact on the conclusion from a group comparison by comparing DTI and DKI parameters between PDD patients and healthy controls.

## Methods

### Image acquisition

Multi-shell diffusion MRI data were acquired in a group of patients with PDD (mean age ± SD; 74±7 years, n = 11) and age-matched controls (age 70±4 years, n = 27). Imaging was performed on a Siemens Skyra 3T scanner equipped with a 32 channel head coil. The diffusion MRI protocol comprised 99 DWI volumes, where the choice of b-values and encoding directions was inspired by Poot et al [18]. Three volumes were acquired with *b* = 0 s/mm^2^, and the remaining volumes were acquired using b-values of 250, 500, 1000 and 2750 s/mm^2^ distributed over 6, 6, 20, and 64 directions, respectively. A single-shot spin-echo with EPI read-out was used, with the following settings: TR = 8100 ms, TE = 103 ms, voxel size = 2.3×2.3×2.3 mm^3^, FOV = 294×294 mm^2^, iPAT = 2, and partial Fourier factor = 6/8. The imaging volume comprised 52 contiguous axial slices adjusted to include the whole cerebrum. Total acquisition time was 14 minutes.

### Ethics statement

The study, including the consent procedure, was approved by the Regional Ethical Review Board (“Regionala Etikprövningsnämnden i Lund”), Lund, Sweden (number 2011–277), and was performed in accordance with the Helsinki Declaration. Patients and healthy controls were informed of the study content in both oral and written form. Informed consent was taken in written form. All participants were judged to have the capacity to consent, gave written informed consent prior to participation, and underwent cognitive testing and neurological examination by a medical doctor. Patients diagnosed with PDD met the Clinical Diagnostic Criteria for Dementia Associated with PD according to Emre et al [19]. Controls exhibited no parkinsonian symptoms or cognitive deficits.

### Conventional motion and eddy-current correction

Conventional motion and eddy-current correction was performed by registering each volume to the first volume acquired with *b* = 0 s/mm^2^. The registration utilized an affine transformation with 12 degrees of freedom [12], and was performed using ElastiX [20]. As the objective function, we employed mutual information, and the final image was interpolated with a b-spline of order 3. The Jacobian determinant of the affine transformation matrix was applied to rescale the image intensity [12].

For completeness, we also performed conventional correction using the ‘eddy_correct’ tool in FSL, which registers the DWI volumes to the first volume acquired with b = 0 s/mm^2^, using an affine transform implemented in FLIRT (www.fmrib.ox.ac.uk/fsl).

### Extrapolation-based correction

**Fig 2:**
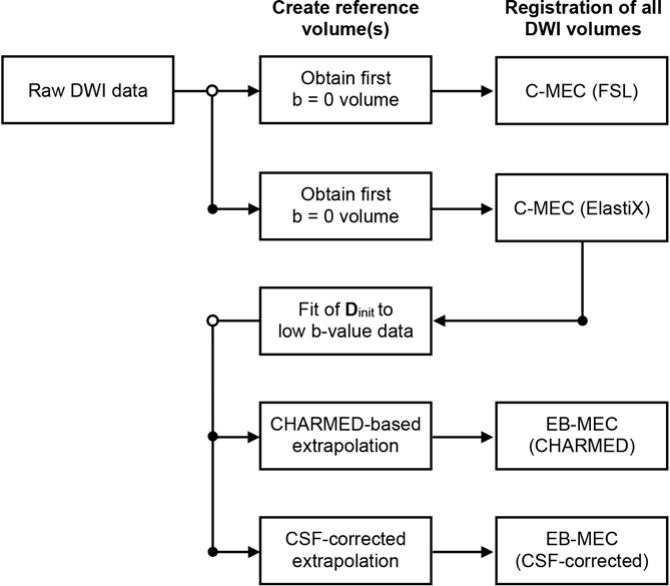
Flowchart of the motion and eddy-current correction (MEC) procedures. Two conventional registration procedures (C-MEC) utilizing different registration softwares were explored, and two extrapolation-based correction procedures (EB-MEC) using different extrapolation techniques.

Extrapolation-based correction was performed by registering each volume to a reference extrapolated from a model fitted to low b-value data that had first been corrected using conventional correction. The registration employed the same settings as for the conventional correction with ElastiX. Two different extrapolation methods were investigated, as described below. Fig 2 shows a flowchart of the correction procedures.

Each set of reference volumes was extrapolated from low b-value data (b ≤ 1000 s/mm^2^). As a starting point for the extrapolation, we used DTI defined by

$$S(B)=S_{0}\;\text{exp}(-<B,D_{\text{init}}>),$$ (1)

where **B** is the b-matrix, ‘< ·, · >’ denotes the tensor inner product, and **D**_init_ is the initial tensor resulting from the model fit. In previous work, the diffusion tensor model itself has been used to interpolate references for low b-value shells [21]. This approach is, however, not suitable for extrapolation of high b-value references since DTI is not accurate for b-values above approximately 1000 s/mm^2^. Here we investigate the use of the CHARMED model for extrapolation, as proposed by Ben Amitay [16], and a novel model where we correct for partial volume effects from CSF, as CSF is known to corrupt diffusion tensor estimates [22]. Other procedures that have been explored for creating references include, for example, averaging of the diffusion-weighted images in each shell [23] the use of CSF suppression by fluid attenuated inversion recovery (FLAIR) [24], were not included in this investigation.

#### CHARMED-based extrapolation

Extrapolation of references for registration has previously been performed using the CHARMED model [16]. This model is based on two tensors, modelling hindered and restricted diffusion, with signal fractions given by *f*_r_ and *f*_h_ (see Appendix 1 for the full model). The value of *f*_r_ can be approximated from the fractional anisotropy (FA) of **D**_init_ according to *f*_r_ = FA/0.78 and *f*_h_ from *f*_h_ = 1 –*f*_r_ [16]. Calculation of the tensor for the restricted component requires an estimate of the axon radius, but how to calculate this parameter from **D**_init_ is not fully specified in [16]. We therefore assumed the effective radius to be zero, which is a reasonable assumption for most of the white matter and most experimental protocols [7,25]. The restricted tensor **D**_r_ was thus calculated according to **D**_r_ = AD_r_ **uu** ^T^, where AD_r_ = 1.7 μm^2^/ms is the axial diffusivity of the restricted component, and **u** is a column vector of unity length that was obtained from the principal diffusion direction of **D**_init_. The hindered tensor was approximated by the initial tensor, **D**_h_ = **D**_init_, according to [16].

**Fig 3:**
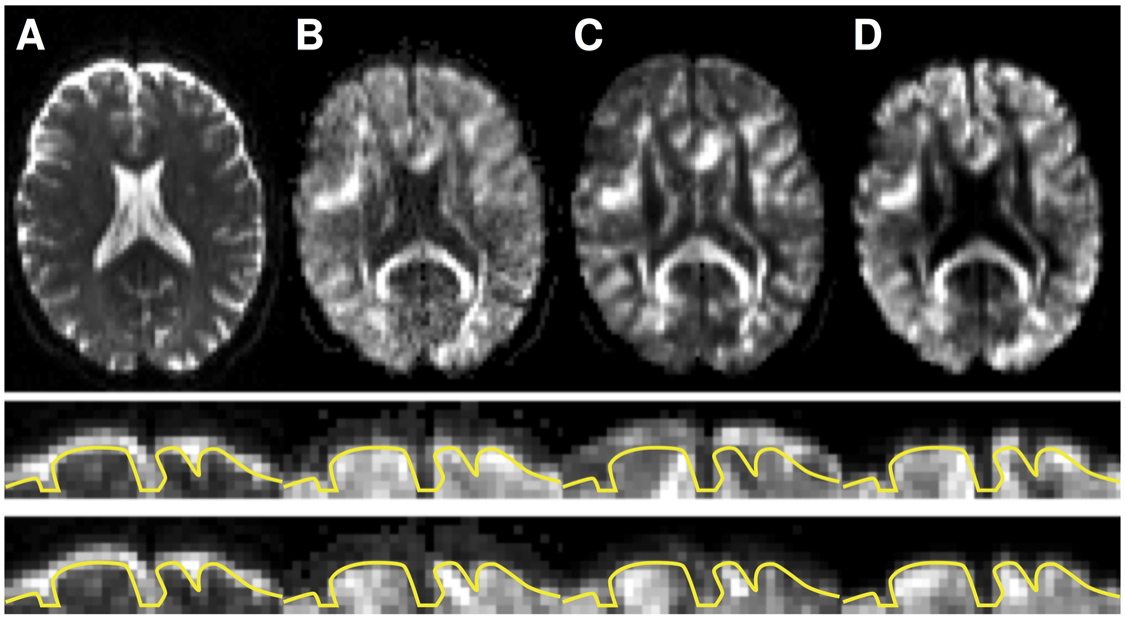
Comparison of image contrasts. A: Acquired without diffusion encoding. B: Acquired using a high b-value. C: Extrapolated using the CHARMED model. D: Extrapolated using the proposed method with CSF correction. The two bottom rows show magnifications of anterior segments of the brain for two diffusion encoding directions. The yellow lines show the outline of the brain at a fixed position across all of the images. Note how the anterior rim of CSF is completely attenuated in the high b-value image (compare A and B). Both extrapolation methods yielded images with gross contrast similar to the acquired images. However, the CHARMED model introduced a shift in the outline of the brain for some encoding directions (compare outlines between bottom two rows in column C). For the proposed method, the outline of the brain did not vary substantially with encoding direction (column D).

Using this model for extrapolation, we found that the edge between extrapolated signal and background depended on the diffusion encoding direction (compare Fig 3B and 3C, two lower rows), in particular where the CSF-filled subarachnoid space is wide. The dependency was caused by an assumption in the CHARMED model that enforced highly anisotropic diffusion of the slowly diffusing component. This assumption is, however, not valid in grey matter where the voxel-scale diffusion appears nearly isotropic. Another reason for the discrepancy is that in the cortical GM region, the signal from tissue is mixed with that from CSF. This partial-volume effect is not accounted for in the initial DTI fit, and resulted in an extrapolation with lower image intensity than expected.

#### CSF-corrected extrapolation

To improve the signal prediction, we developed an extrapolation approach that corrected for partial volume effects from CSF and that did not assume high anisotropy by default. This resulted in references more similar to the acquired data (compare Fig 3B and 3D).

Partial volume correction for CSF was performed by separating the initial diffusion tensor **D**_init_ into two components representing tissue and CSF. Assuming an isotropic tensor for CSF with a diffusivity of *D*_CSF_, we calculated the tissue tensor **D**_tissue_ from the relation

$$D_{\text{init}}=(1-f_{\text{CSF}})D_{\text{tissue}}+f_{\text{CSF}}D_{\text{CSF}}I,$$ (2)

where **D**_init_ is obtained from fitting the tensor to the low b-value shell, **I** is the unit tensor, and *f*_CSF_ is the signal fraction of CSF. Three assumptions were required to solve this equation for **D**_tissue_ and *f*_CSF_. First, we assumed *D*_CSF_ = 2.1 μm^2^/ms. This value is lower than the expected value of 3 μm^2^/ms, but we found that a lower value made the procedure more robust. Second, we assumed all tissue to have equal mean diffusivity, i.e., that Tr(**D**_tissue_)/3 = *D*_tissue_ = 0.8 μm^2^/ms, where Tr(·) denotes the trace. This assumption is reasonable in both white and grey matter where the mean diffusivity is in the range 0.8–0.9 μm^2^/ms during adulthood [26,27]. Third, we assumed that *f*_CSF_ can be approximated from

$$\frac{1}{3}\text{Tr}\left(D_{\text{init}}\right)=f_{\text{CSF}}D_{\text{CSF}}+(1-f_{\text{CSF}})\frac{1}{3}\text{Tr}\left(D_{\text{tissue}}\right).$$ (3)

Values of *f*_CSF_ were then constrained to the interval from zero to unity, after which **D**_tissue_ was calculated from Eq 2. Note that the values on *f*_CSF_ obtained through this procedure are not appropriate for any quantitative comparisons [22], and are thus only used in the extrapolation procedure. A second correction of **D**_tissue_ was introduced to increase the accuracy of the extrapolation; voxels with a mean diffusivity below *D*_min_ = 0.3 μm^2^/ms were modified according to **D**′_tissue_ = **D**_tissue_ + *h*(**D**_tissue_), where *h*(**D**_tissue_) is a function given by

$$h\left(D_{\text{tissue}}\right)=\left\{ \begin{array}{l} \left(D_{\text{min}}-\frac{1}{3}\text{Tr}\left(D_{\text{tissue}}\right)\right)I\;\text{if}\;\frac{1}{3}\text{Tr}(D_{\text{tissue}})<D_{\text{min}}\\ \;0\;\text{otherwise} \end{array}\right.$$ (4)

To avoid introduction of artificial diffusional anisotropy in grey matter, we based the extrapolation solely on **D**′_tissue_. To model slow diffusion, we employed the stretched-exponential model for the extrapolation [28]. This model is based on the assumption that there is a distribution of diffusion coefficients present in the voxel, where the width of the distribution is modulated by the parameter α. We believe this model to be useful for extrapolation, since there is pronounced orientation dispersion in most voxels of the brain, and orientation dispersion induce a rather wide distribution of diffusion coefficients [29]. We fixed α to 0.8, which is representative for cortex, but slightly high for white matter [28]. The final model was given by

$$S\left(B\right)=S_{0}\left(\left(1-f_{\text{CSF}}\right)\text{exp}\left(-<B,D_{\text{tissue}}^{\prime }{>}^{\alpha }\right)+f_{\text{CSF}}\;\text{exp}\left(-<B,D_{\text{CSF}}>\right)\right).$$ (5)

### Comparing correction results

Both the CHARMED model and the CSF-corrected extrapolation model were used to extrapolate reference volumes for all DWI volumes acquired, which were then used for motion and eddy-current correction (see flowchart in Fig 2).

To determine the accuracy of the motion and distortion correction procedures, we calculated FA maps from both the low and the high b-value part of the data and registered the latter to the former using an affine transform. Our hypothesis was that a bias-free correction should result in zero translation, shearing, rotation, and scaling between the two FA maps. We assumed that the overall contrast of the FA maps, important to the registration, were robust to slight misregistrations within the low and the high b-value subset.

We also analysed the time series of the transform parameters from the corrections, averaged across all subjects. For each volume acquired, the affine registration yields parameters associated with translation, rotation, scaling, and skewing along and around the x, y, and z axes (*a*_i_, *i* = 1–12). Some of the registration parameters depend on the eddy-currents, which in turn depend on the diffusion-encoding gradient, **g** = (*g*_x_, *g*_y_, *g*_z_) [30]. In order to assess the degree to which eddy-currents may explain variation in these parameters, they were each modelled as a linear function of **g** for volume *j* according to [30]

$$a_{i}\left(j\right)=\beta_{0}+\beta_{1}g_{x}\left(j\right)+\beta_{2}g_{y}\left(j\right)+\beta_{3}g_{z}\left(j\right),$$ (6)

where *β*_0_, *β*_1_, *β*_2_, and *β*_3_ can be obtained by linear regression. We performed the regression and determined the coefficient of determination (R2) that represents the amount of variation between volumes explained by the regression. High values of R2 indicate that variation in transform parameters are explained by eddy-currents. Low values of R2 are expected if eddy-currents do not affect the transform parameter, which should be the case e.g. for rotations around and translations along the x and z-directions [30].

### DTI and DKI analysis

Diffusion data were used to calculate FA-maps by fitting the DTI model to the low b-value data (b ≤ 1000 s/mm^2^). Maps of the mean diffusivity (MD) and the mean of the kurtosis tensor (MW) were obtained by fitting the following model to the geometrical average of the MR signal from the different shells [31,32]

$$S(b)=S_{0}\left(\left(1-f_{\text{CSF}}\right)\text{exp}\left(-b\;\text{MD}+\frac{1}{6}{\text{b}}^{2}{\text{MD}}^{2}\text{MW}\right)+f_{\text{CSF}}\;\text{exp}\left(-b\;D_{\text{CSF}}\right)\right),$$ (7)

where *S*_0_, MD, MW and *f*_CSF_ were free variables and *D*_CSF_ was fixed to 3.1 μm^2^/ms. Note that the model in Eq 7 is an extension of the conventional DKI model that also accounts for partial volume effects from CSF.

In order to assess the effect of the motion and eddy-current correction method on DKI parameters, we obtained histograms of MD, FA, and MW in regions of interest defined to encompass the white matter, i.e. all voxels with FA above 0.4 and where at least 14 out of 26 neighbouring voxels also fulfilled the FA condition.

### Tractography and group comparisons

One of the main objectives of this study was to evaluate the effect of the motion and eddy-current correction method on the outcome of a group comparison. For this purpose, we performed HARDI tractography to extract the cingulum and the anterior thalamic projection tracts, and compared DTI and DKI parameters between the PDD patients and the healthy controls. Our choice of tracts was inspired by a study where DKI of the cingulum bundle was suggested as a means to improve the ability to diagnose Parkinson’s disease [33].

After motion and eddy-current correction, the high b-value shell with 64 encoding directions was processed using MRtrix [34]. The analysis employed constrained spherical deconvolution (CSD) to model multiple fibre orientations in each voxel, with a maximum harmonic order of 8 [35]. Probabilistic fibre-tracking was performed using a step-size of 0.5 mm, and terminated in regions where FA was below 0.1.

Three segments of the cingulum were extracted according to the scheme proposed by Jones et al [36], i.e., the subgenual, retrosplenial and parahippocampal segments. The tract segments were cropped so that only well-specified parts were retained. The subgenual streamlines were cropped at the level of the inferior genu and at the middle of the corpus callosum. Retrosplenial tracts were cropped at the middle of the corpus callosum and at the inferior border of the splenium. Parahippocampal segments were cropped at the inferior border of the splenium and at a level corresponding to the middle of the pons. Tracts projecting anteriorly from the thalamus were also extracted, followed by a cropping procedure that removed segments of the tracts entering the thalamus or proceeding anteriorly to the genu of the corpus callosum. In all cases, regions of interest (ROIs) were placed in MNI-space and projected back to native space using warp fields calculated by FNIRT (www.fmrib.ox.ac.uk/fsl) [37], applied to the FA volumes. Seeding was performed in a region fully encompassing the region in which tracks were expected to be found in order to address the path-length dependency of tractography [38].

Quantification of DKI parameters was adapted to minimize the influence of partial volume effects from CSF. We calculated the weighted parameter average for each streamline in the tract, according to

$$y=\frac{\sum_{i}w_{i}y_{i}}{\sum_{i}w_{i}},$$ (8)

where *y* is the parameter of interest and *w*_i_ = 1 –(*f*_CSF_)_i_ with *f*_CSF_ obtained from Eq 7, and *i* index positions along the tract. This procedure was most important in the thalamic projection tracts, which pass close to the ventricles, and occasionally included streamlines passing through voxels with a high CSF content where the parameter values were less well determined than in pure WM. Values of FA, MD and MW were then averaged across all streamlines, and tested for differences between controls and patients with PDD while correcting for age using linear regression, implemented in MATLAB (MathWorks, Natick, MA). The significance threshold was set to 0.05.

## Results

### Accuracy of the correction

**Fig 4:**
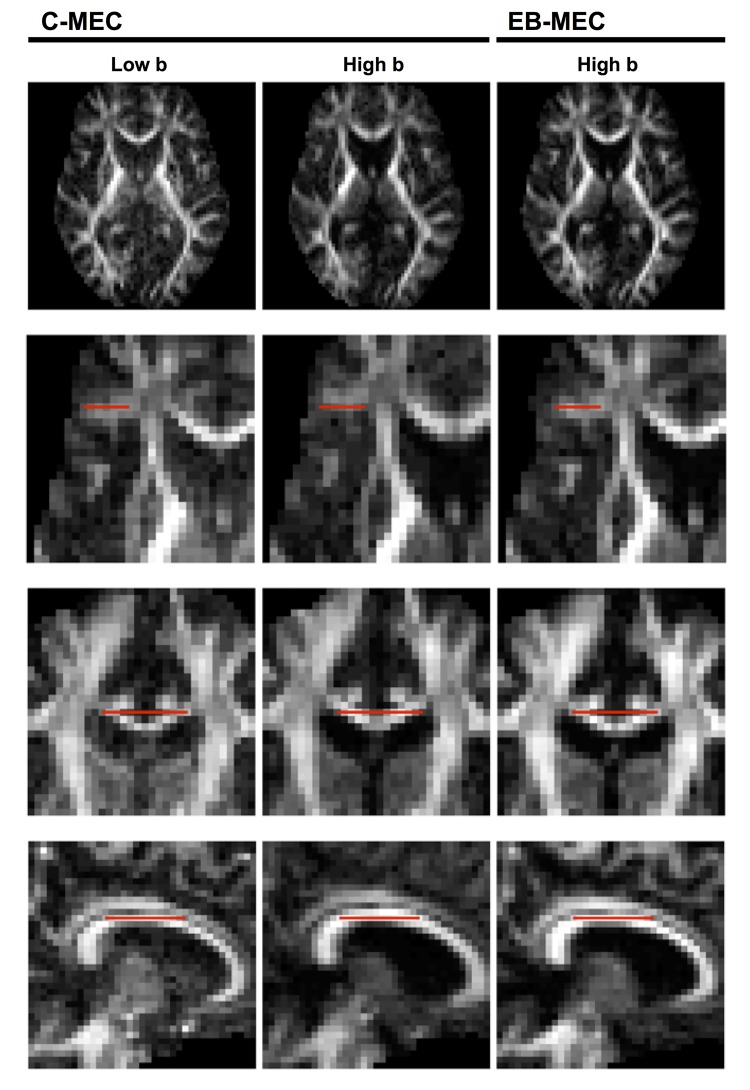
Illustration of the registration error between low and high b-value volumes. The left and middle columns show FA-volumes obtained from a low and a high b-value data set, respectively, processed using the conventional motion and eddy-current correction method (C-MEC). The mismatch between the two data sets becomes apparent when the position of the corpus callosum and surrounding tracts (second, third, and fourth row) are compared to a fixed position indicated by the red line. The third column shows FA projections from high b-value data processed using the CSF-corrected extrapolation-based motion and eddy-current corrections (EB-MEC), where no apparent mismatch between the low and high b-value volumes is visible.

The accuracy of conventional and extrapolation-based correction was evaluated by comparing FA maps calculated from the low and high b-value data. Fig 4 shows three sets of FA maps based on (i) conventional correction of the low b-value data set, (ii) conventional correction of the high b-value data set, and (iii) extrapolation-based correction of the high b-value data set. While the overall contrast in the FA maps did not differ much between these maps, substantial shifts of up to two voxels were observed between the FA map from the low and high b-value data set when using conventional correction. This discrepancy was quantified by registering the FA volumes from the high b-value data set to those from the low b-value data set (Table 1). The conventional approach resulted in substantial translation and scaling regardless of whether the registration was performed with ElastiX or FSL. For data corrected using extrapolated references, the average transform parameters were close to the expected values of zero for both methods, except for a scaling of approximately 1% along the z-direction when using the CHARMED model for extrapolation.

**Table 1:** Accuracy of motion and eddy-current correction methods. The table shows mean (standard deviation) of rotation, translation, scale and skew parameters obtained by registering FA-volumes calculated using high b-values to those calculated using low b-values. Top two sub tables show results from conventional motion and eddy-current correction (C-MEC) using FSL and ElastiX whereas the bottom two subpanels show results from CSF-corrected and CHARMED-based extrapolation-based motion and eddy-current correction (EB-MEC).

**C-MEC (FSL)**	**Axis: X**	**Axis: Y**	**Axis: Z**
**Rotation (deg)**	–0.05 (0.8)	0.10 (0.3)	0.12 (0.4)
**Translation (mm)**	–0.06 (0.3)	0.99 (0.5)	0.94 (0.7)
**Scale (%)**	1.66 (1.0)	1.71 (0.8)	1.40 (1.0)
**Skew (%)**	0.01 (0.6)	-0.01 (0.5)	–0.27 (0.9)
**C-MEC (ElastiX)**	**Axis: X**	**Axis: Y**	**Axis: Z**
**Rotation (deg)**	0.00 (0.4)	0.00 (0.2)	0.14 (0.3)
**Translation (mm)**	–0.59 (0.4)	1.07 (0.8)	0.55 (0.7)
**Scale (%)**	1.14 (1.3)	2.76 (1.3)	2.56 (2.1)
**Skew (%)**	–0.09 (0.3)	–0.12 (0.5)	–0.21 (0.7)
**EB-MEC (CSF corrected)**	**Axis: X**	**Axis: Y**	**Axis: Z**
**Rotation (deg)**	–0.08 (0.2)	–0.01 (0.2)	0.04 (0.1)
**Translation (mm)**	–0.01 (0.2)	0.13 (0.1)	–0.11 (0.2)
**Scale (%)**	0.65 (0.2)	0.71 (0.3)	0.35 (0.6)
**Skew (%)**	0.02 (0.3)	0.01 (0.2)	–0.19 (0.4)
**EB-MEC (CHARMED-based)**	**Axis: X**	**Axis: Y**	**Axis: Z**
**Rotation (deg)**	–0.08 (0.2)	–0.02 (0.2)	0.02 (0.1)
**Translation (mm)**	–0.02 (0.1)	0.17 (0.2)	–0.12 (0.2)
**Scale (%)**	0.77 (0.2)	0.74 (0.3)	1.04 (0.4)
**Skew (%)**	–0.04 (0.2)	0.04 (0.2)	–0.22 (0.3)

**Fig 5:**
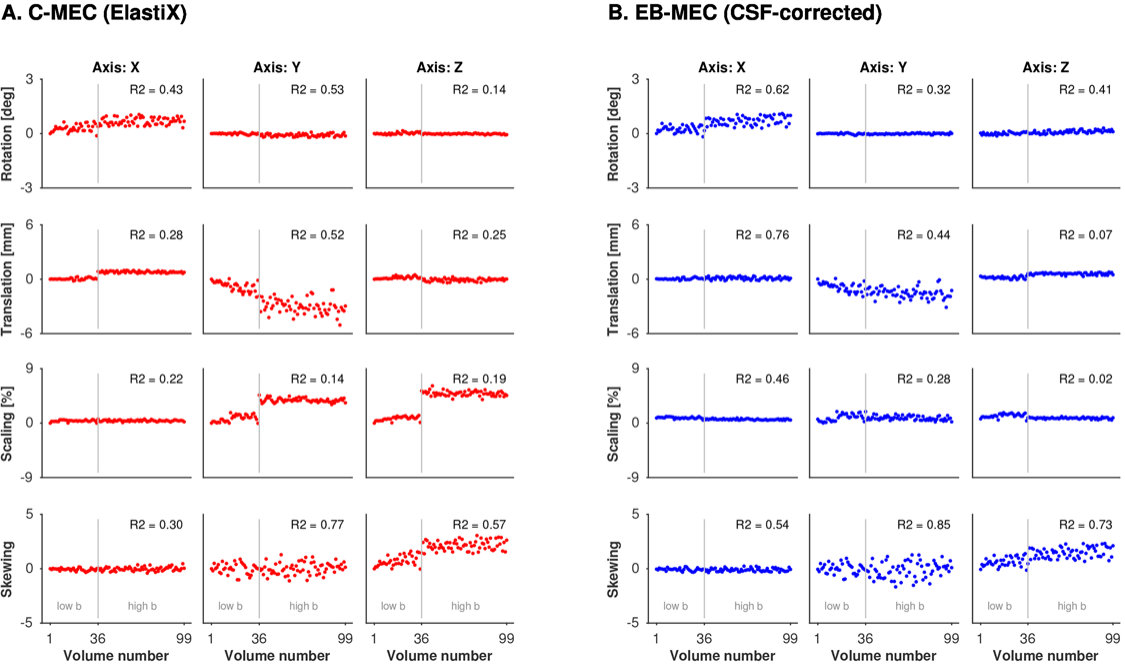
Transform parameters from the motion and eddy-current correction versus volume number. Data were averaged across all subjects, for the conventional correction (A, red) and the CSF-corrected extrapolation-based procedure (B, blue). Rows and columns show values of the rotation, translation, scaling and skewing parameters around and along the x, y, and z axes, respectively. Deviations from zero can be expected, for example, due to eddy-currents or misregistration. The value of R2, i.e., the amount of variability explained by regressing gradient amplitudes onto the data, is shown in the top right corner of each panel. High values of R2 indicate that eddy-currents explain much of the variation in the transform parameters. Acquisition of high b-values started at volume number 36, which explains the discontinuity of, for example, the z-scaling in the C-MEC data.

The superior performance of the extrapolation-based methods is also supported by inspection of the translation, rotation, scaling and skew parameters, averaged across all subjects (Fig 5 and S1 Fig). Nonzero values can be due to eddy-currents, subject motion, or errors caused by the registration. Parameters correcting only for subject motion, i.e., rotations and translations in the x- and z-directions, respectively, were expected to average to zero in the population. By contrast, most parameters from the conventional methods showed substantial shifts from zero in the high b-value volumes (Fig 5 and S1 Fig, red dots). Parameters from the extrapolation-based corrections (Fig 5 and S1 Fig, blue dots), however, followed expectations and were scattered around zero for all except four parameters: rotation around the x-axis, translation along the y-axis and skew along the y and z-axis. The variation in these parameters were generally explained well by regressing the gradient amplitudes onto the data, where R2 was 0.62, 0.44, 0.85 and 0.73 for the CSF-corrected extrapolation method, and 0.64, 0.40, 0.87, and 0.67 for the CHARMED-based method. Due to the high values of R2, the variations can be credibly attributed to true eddy-current effects. A notable difference between the CSF-corrected and the CHARMED-based methods was that the high b-value volumes from the CHARMED-based correction were on average scaled by 2.2% in the z-direction, compared to 0.8% for the volumes corrected with CSF-corrected extrapolation. This difference in scaling was visible in both references and corrected images (S2 Fig).

### Effects on DKI parameters

**Fig 6:**
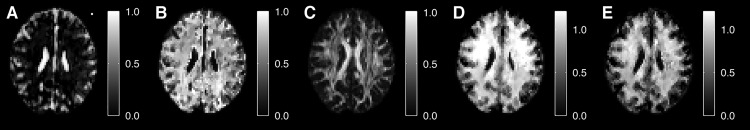
Parameter maps from the model fitting. Panel A shows the fraction of CSF (*f*_CSF_ from Eq 7). Panel B shows the mean diffusivity (MD) weighted by *w* = 1–*f*_CSF_. Panel C shows the fractional anisotropy (FA). Panel D and E show the mean kurtosis (MW) weighted by *w*, estimated from data corrected using the CSF-corrected extrapolation-based, and the conventional method, respectively.

**Fig 7:**
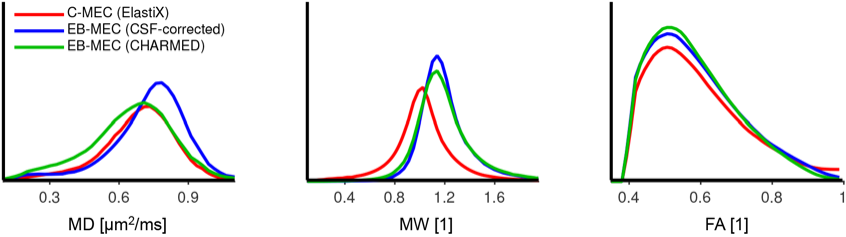
Histograms of DKI and DTI parameters in white matter. The lines represent the conventional correction method (red), extrapolation-based method using the CSF-corrected approach for extrapolation (blue), and the extrapolation-based method using CHARMED for extrapolation (green). With the conventional approach, there was a clear negative bias in the mean kurtosis, MW, but also a large fraction of approximately 10% of all voxels where FA was equal to one (spike not shown in histogram).

Fig 6 shows an example of parameter maps from fitting of the DKI model with CSF-correction (Eq 7). Notably, MW was clearly higher in the data corrected using the extrapolation-based method compared to the conventional method. We believe this discrepancy can be attributed to the erroneous scaling of high b-value volumes by the conventional method (based on ElastiX). This effect is confirmed in Fig 7, which shows histograms of MD, MW and FA in the white matter for the conventional method and the two extrapolation-based methods. Again, MW is clearly higher for the two extrapolation-based approaches, and slightly higher for the CSF-based approach than for the CHARMED-based approach. For MD, the CHARMED-based approach yield lower values than the CSF-based approach.

### Effects on tractography

**Fig 8:**
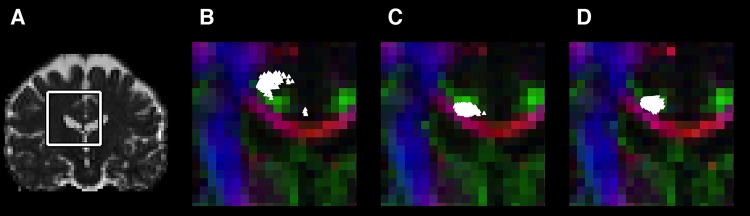
Effect of the registration error on tractography based on high b-value data. Panel A shows the mean diffusivity map of a patient with atrophy. Panels B–D shows a zoomed in section, delineated in panel A. Point clouds in white represent a coronal cross section of the retrosplenial cingulum obtained from tractography using high b-value HARDI data. The tract points are overlaid on top of a colour FA-volume, which was calculated from data acquired with low b-values. When using data corrected with the conventional method (panel B), the point cloud appears in a region approximately two voxels above the expected region (green voxels). Data corrected with the CHARMED-based extrapolation method resulted in a point cloud slightly below the expected region (panel C). For data processed with the CSF-corrected extrapolation-based motion and eddy-current correction the point cloud corresponds well to the anatomical structure (panel D).

The effect of misregistration of high b-value volumes was examined by visualising a coronal cross section of the retrosplenial part of the cingulate bundle obtained from high b-value tractography (Fig 8A). The conventional correction (using ElastiX) produced tracks that exhibited a gross misalignment between the tracks and the expected position of the cingulate bundle (Fig 8B). The tracts were closer to the expected position for data corrected with the CHARMED-based extrapolation method (Fig 8C), but only the CSF-corrected extrapolation-based correction produced tracks that were reliably confined within the intended structure (Fig 8D).

### Effects on group comparisons

**Fig 9:**
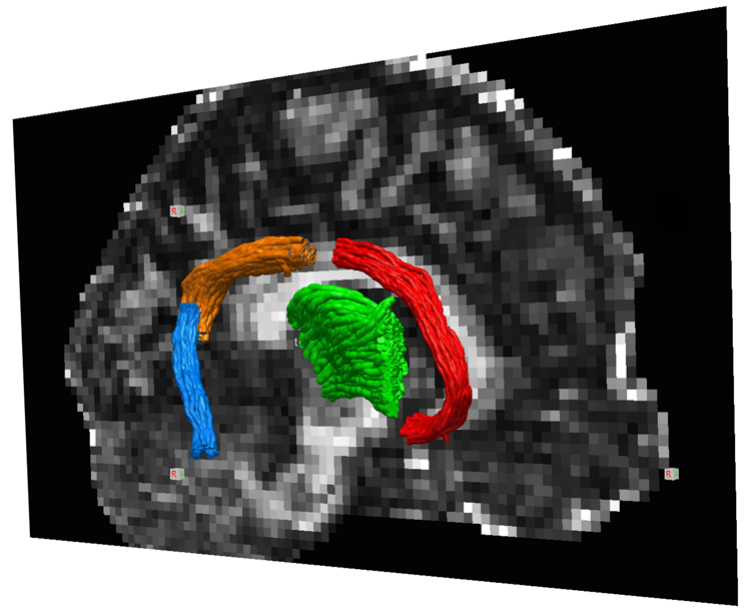
Tractography of the analysed WM bundles. The three segments of the cingulum, i.e., the parahippocampal, retrosplenial and subgenual segment, are shown in blue, orange and red, respectively. Tracks projecting anteriorly from the thalamus are shown in green.

**Fig 10:**
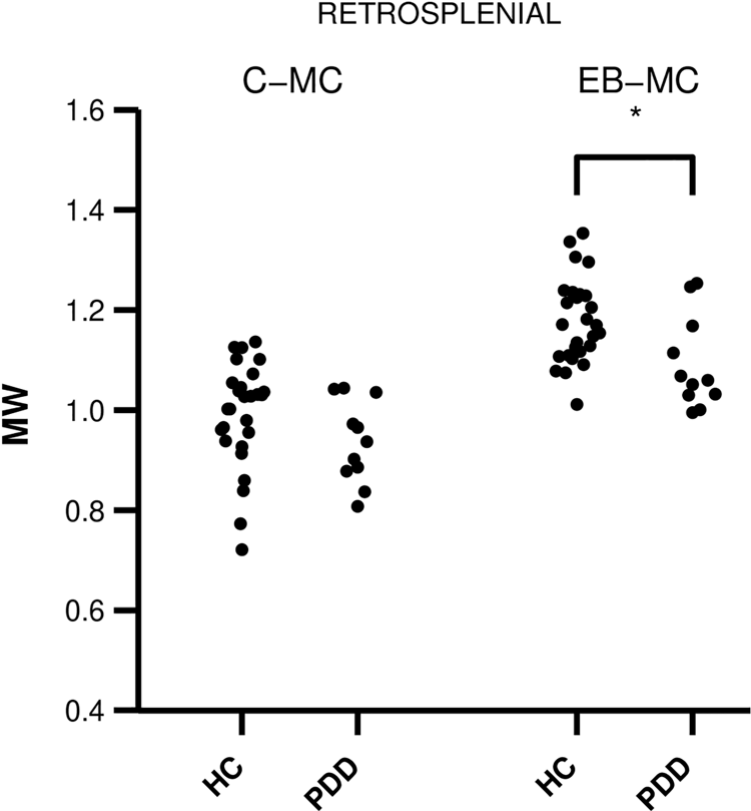
Mean kurtosis (MW) in the retrosplenial segment of the cingulum. The swarm plots show parameters obtained using the conventional and extrapolation-based methods in the healthy controls and PDD patient group. A significant difference was observed for the CSF-corrected extrapolation-based method (*p* = 0.018), which vanished for the data corrected using the conventional method (*p* = 0.84). The extrapolation-based method also resulted in higher values of MW.

The main objective of this study, to assess the impact of the choice of motion and eddy-current correction method on group comparisons, such as comparing a disease group to controls, was pursued by comparing DKI parameters between PDD patients and healthy controls in the tracts shown in Fig 9. When data were corrected using the conventional method (based on ElastiX), significant differences were detected only for FA within the parahippocampal part of the cingulum (Table 2). However, when data was corrected using the CSF-corrected extrapolation-based method, FA differed significantly between the diagnostic groups in the retrosplenial part of the cingulum, and MW differed significantly in the thalamic projection tracts and in the retrosplenial part of the cingulum (Table 2, Fig 10). In this aspect, the CHARMED-based method and the CSF-corrected method yielded similar results. The increase in sensitivity can be explained by increased statistical power mediated by a reduction in parameter variability, in particular for FA and MW when using extrapolation-based registration; the standard deviation of MW was reduced by up to 50% in the subgenual part of the cingulum in the PDD patients. The FA and MW were also generally higher in the data corrected using the extrapolation-based method.

**Table 2:** Effect of motion and eddy-current correction method on group comparison. Results are presented as mean (standard deviation), and were for the left pair of columns obtained using conventional motion and eddy-current correction (C-MEC) whereas the right pair of columns show values obtained using extrapolation-based motion and eddy-current correction. Significant differences were observed more frequently between the healthy controls and PDD patients when using the extrapolation-based correction. Results were similar but not identical when each hemisphere was compared separately.

		C-MEC (ElastiX)			EB-MEC (CSF-corrected)	
		HC (n = 27)	PDD (n = 11)		HC (n = 27)	PDD (n = 11)
Thalamic	MD	0.74 (0.06)	0.79 (0.08)		0.76 (0.05)	0.80 (0.08)
	MW	0.98 (0.13)	0.93 (0.12)		1.17 (0.09)	1.09 (0.08) *
	FA	0.36 (0.04)	0.37 (0.05)		0.47 (0.04)	0.45 (0.04)
CB: Subgenual	MD	0.77 (0.06)	0.75 (0.05)		0.77 (0.05)	0.77 (0.05)
	MW	0.68 (0.12)	0.63 (0.15)		0.91 (0.10)	0.82 (0.09)
	FA	0.26 (0.05)	0.26 (0.06)		0.37 (0.03)	0.36 (0.03)
CB: Retrosplenial	MD	0.71 (0.05)	0.74 (0.05)		0.76 (0.04)	0.76 (0.07)
	MW	0.99 (0.10)	0.93 (0.09)		1.18 (0.08)	1.09 (0.09) *
	FA	0.40 (0.04)	0.39 (0.07)		0.46 (0.03)	0.48 (0.05) *
CB: Parahippocampal	MD	0.64 (0.05)	0.63 (0.07)		0.67 (0.05)	0.66 (0.07)
	MW	0.90 (0.18)	0.84 (0.23)		1.09 (0.13)	1.06 (0.20)
	FA	0.31 (0.04)	0.34 (0.05) **		0.38 (0.04)	0.42 (0.05) **

* p < 0.05

** p < 0.01 (multivariate analysis correcting for age)

## Discussion

Conventional motion and eddy-current correction, where each diffusion-weighted volume is registered to a non diffusion-weighted reference, suffers from poor accuracy for high b-value data. We show that motion and eddy-current correction of high b-value data can be improved significantly by extrapolating reference images from low b-value data. In group comparisons, improved correction resulted in reduces variability in the DKI metrics and may thus lead to increased statistical power and thereby a higher sensitivity for detection of group-wise differences.

We also compared two methods for extrapolation, where one was similar to that of Ben-Amitay et al. [16] and based on the CHARMED framework [39], and the other was a novel approach where we corrected for partial volume effects with CSF (Eqs 2–4). The overall results were similar, but correction for CSF yielded registrations that were visible improved (Table 1, Fig 8 and S2 Fig). The CSF corrections assume prior knowledge of the mean diffusivity of tissue and CSF, which can be readily estimated from data or found in the literature. Although these priors may be different in, for example, tumours, ischemia, or neurodegeneration, we do not expect the method to be overly sensitive to such deviations due to the use of mutual information in the image registration, which makes it robust to local deviations in image contrast.

The main reason why extrapolation-based methods can be expected to be superior to conventional methods is the substantially different contrast in high b-value versus non diffusion-weighted volumes (Figs 1 and 3), as also reported by other groups [16,40]. For low b-values, all methods apart from the FSL-based conventional correction performed similarly well (e.g., compare the parameters for low b-values in Fig 5 and S1 Fig), probably because the CSF signal is not fully attenuated and thereby represented in the mutual information. However, benefits of using model-based references also for low b-values have been reported [21]. At higher b-values, CSF is fully attenuated; the MR-signal in CSF at *b* = 0, 1000 and 3000 s/mm^2^ is 100%, 5%, and 0.01%. Corresponding values in WM, assuming encoding perpendicular to the structure, are 100%, 90%, and 70%. Even at *b* = 28000 s/mm^2^, approximately 20% of the WM signal can remain [41]. This indicates that regardless of whether the image comparison metric takes contrast differences into account or not, adequate alignment of the rim of CSF around the brain is virtually impossible in high b-value volumes where the CSF signal is completely attenuated (Fig 1).

We would like to emphasize that optimal motion and eddy-current correction of DWI data is a question open to discussion and further research. For example, the use of a global affine transformation neglects slice-to-slice variations of the eddy-current distortions [11]. However, affine registrations have been reported to be sufficient for whole-brain correction [30]. Another aspect of correction that we, and others, neglect is the interaction between susceptibility distortions and motion. This may result in additional smoothing because the effective direction in which the susceptibility effects distort the image will depend on rotations of the head. Optimal correction of susceptibility distortion requires that data is acquired with reversed polarity of the phase encoding gradients in the EPI sequence [42]. For such data, and with gradient directions spread out across the whole sphere, a Gaussian-process approach has shown promising results [43]. Unfortunately, standard acquisition protocols yield data unsuitable for this approach since the EPI is acquired with unipolar phase encoding gradients and gradient directions are spread out across a half sphere only (e.g. see instructions for ‘eddy’ from FSL). Addressing the motion and eddy-current correction of high b-value data acquired with standard protocols is important because high b-value diffusion data are gaining popularity, warranting more research into this topic. Here, we show that the step from conventional to extrapolation-based correction is of utmost importance for the accuracy of the registration (Table 1), while the benefit of improving the extrapolation method (CSF-corrected vs CHARMED) is moderate but visible (Fig 8 and S2 Fig), and possibly important e.g. for cortical analyses.

Determining the quality of a registration is a non-trivial task, as discussed in the context of non-rigid registrations by Crum et al. [44]. We found that simple visual inspection of the DWI series as a cine loop was not sufficient to detect the subtle shifts reported in Table 1. Inspection of the average registration parameters may raise suspicion of suboptimal registration. For example, the y and z scaling for high b-value image volumes in Fig 5A appear unreasonable, but such an observation cannot singlehandedly be considered as proof of erroneous registration. A quantitative test of the accuracy of the registration can, however, be performed by registering FA volumes calculated from high b-value data to those calculated from low b-values (Table 1) because of the high contrast similarity in the two images (Fig 4). This comparison revealed a substantial scaling of the high b-value volumes, for data corrected using the conventional method, of up to 2.8%, while the CSF-corrected extrapolation-based correction reduced the erroneous y-scaling to 0.7%. In the z-direction, the CHARMED-based method resulted in a z-scaling of 1.0%, compared to 0.4% for the CSF-corrected method, likely due to the accumulation of CSF in superior part of the subarachnoid space and the smaller field of view in the z-direction compared to the y-direction (compare Fig 2 and S2 Fig). While both extrapolation-based methods generally yielded superior results compared to the conventional methods, some residual errors were observed. For example, high b-value volumes were unexpectedly rotated by approximately 1 degree around the x-axis (Fig 5B, top left plot). A potential explanation is the difficulty to distinguish a weak skew from a small rotation.

Erroneous scaling and rotation may cause secondary errors. True eddy-currents cause scaling of the image volume, which affects the image intensity. This can be corrected for by rescaling the image intensity by the Jacobian determinant of the affine transformation matrix [12]. True rotations are also represented in the transformation matrix, which can be used to correct the b-matrix for improved tractography [12]. Both of these corrections may deteriorate rather than improve the quality of the data, if the eddy-current and motion correction procedure is inaccurate. Erroneous scaling and rotation was most prominent when using the conventional method, but are to a lesser degree also present when using extrapolation-based methods. Scaling errors were also larger for data corrected using the CHARMED-based method compared to the CSF-corrected approach (Table 1). Erroneous image rescaling when using the conventional method may explain the large effect on MW observed in the parameter maps (cf Fig 6D and 6E), since this metric is sensitive to the signal amplitude of high b-value volumes. Table 2 shows an increase in the average MW of approximately 30% and a reduction of its standard deviation of approximately 20% when using the CSF-corrected extrapolation-based method instead of the conventional method. Similar numbers apply for the FA, whereas the effect on MD is negligible. The result for FA and MD is probably due to the misalignment of tracts for the conventional method (Fig 8). Similarly, large effects of tract misalignment may be expected in maps with high contrast within the white matter (FA), but not in maps with low contrast (MD). We expect that the reduced standard deviation in these metrics will yield higher statistical power in group comparisons based on, for example, DKI [45] and filter exchange imaging [46].

The choice of motion and eddy-current correction method can impact the outcome of group comparisons, as we demonstrated by comparing data from patients with PDD to data from healthy elderly controls (Table 2, Fig 10). Elderly subjects tend to have a pronounced rim of CSF around the brain, and the PDD group is expected to exhibit substantial cerebral atrophy [47]. In PDD, alterations in the tissue microstructure have been observed by DTI in the substantia nigra and the putamen [48–51], and in the genu of the corpus callosum, the superior longitudinal fasciculus and the cingulum [49]. The DTI findings have not been substantially lateralised [49,52], which is why we chose to average across the hemispheres. A large fraction of patients with PDD show, in addition to α-synuclein pathology, amyloid pathology similar to that of Alzheimer’s disease (AD), which is associated to structures relevant for episodic memory function such as the hippocampus and the anterior nucleus of the thalamus that connect to the hippocampus via the cingulum [53,54]. Effects of PDD on the anterior thalamic tracts and the retrosplenial part of the cingulum bundle is thus not surprising, but the increase in FA of the PDD patients in the parahippocampal cingulum is unexpected. However, FA is a sensitive but not very specific parameter. Elevated FA has, for example, been reported in AD patients in regions of crossing fibres, due to selective degeneration of one fibre population [55]. In the left parahippocampal cingulum, ageing in an elderly group unexpectedly resulted in elevated FA, which was hypothesized to be the result of reduced sprouting of hippocampal axons [56]. These unexpected findings are the result of inherent limitations of the DTI model, which can be addressed by new imaging protocols that enable imaging of microscopic anisotropy [4,29,57]. Finally, we note that diffusion MRI data should always be interpreted with care, particularly since residual effects of subject motion may yield spurious group differences [58].

## Conclusion

Conventional motion and eddy-current correction, where diffusion-weighted volumes are registered to a non diffusion-weighted volume, is inadequate for high b-value data, due to the fundamentally different contrast between the volumes being registered. In particular, the complete attenuation of CSF signal in volumes acquired with b-values above approximately 1500 s/mm^2^ renders a challenge for the conventional approach. Registration to extrapolated references that have a contrast similar to the acquired data results in substantial improvements in registration accuracy and improved geometrical correspondence between parameter maps based on low and high b-value data. This is of importance for DKI as well as for high b-value tractography. In data processed with the extrapolation-based method, mean kurtosis was approximately 30% higher than in the data processed with the conventional method, probably due to the erroneous scaling induced by the conventional method. The CSF-corrected extrapolation approach yielded more accurate registration results than the CHARMED-based approach. Motion and eddy-current correction has a significant influence on DKI parameters, and thus quantitative comparisons between different DKI studies should take the choice of correction method into account. When applied to a comparison between patients with PDD and healthy controls, the extrapolation-based method yielded a higher statistical power for detecting the diffusion changes, compared to the conventional method. In summary, extrapolation-based methods are strongly recommended for motion and eddy-current correction of high b-value data.

## Appendix

### Definition of the CHARMED model

The CHARMED model is given by Eq 14 in the paper by Assaf et al [59]. The model describes the diffusion in terms of a hindered and a restricted component, denoted by subscripts h and r, respectively. In the notation used herein, we can express the normalized MR signal *E* in terms of two diffusion tensors,

$$E=f_{\text{h}}\text{exp}\left(-<B,D_{\text{h}}>\right)+f_{\text{r}}\text{exp}\left(-<B,D_{\text{r}}>\right).$$

where the fractions are denoted *f*_h_ and f_r_, respectively, and *f*_h_ + *f*_r_ = 1. The b-tensor (**B**) is calculated from the time between the leading edges of the diffusion encoding gradients (Δ), their durations (δ), and the q-vector (**q** = γδ**g/**2π, with γ as the gyromagnetic ratio and **g** as the gradient vector), according to

$$B=4\pi^{2}\left(\Delta -\frac{1}{3}\delta \right)qq^{\text{T}},$$

The diffusion tensor of the extracellular space is given by **D**_h_ and denoted *D* in Eq 10 of [59]. In the intra-axonal space, diffusional displacements are assumed to be restricted by the axonal walls but uncorrelated in different directions [59]. The diffusion can thus be represented by a cylinder-symmetric and time-dependent diffusion tensor, according to

$$D_{\text{r}}={\text{RD}}_{\text{r}}I+\left({\text{AD}}_{\text{r}}-{\text{RD}}_{\text{r}}\right)nn^{\text{T}},$$

where **n** is the direction of the fibre, AD_r_ is the intra-axonal axial diffusivity represented by D_||_ in Eq 7 of [59]. The time-dependent radial diffusivity RD_r_ is given by

$${\text{RD}}_{\text{r}}=\frac{7}{96}\frac{R^{4}}{D_{⊥}\tau }\left(2-\frac{99}{112}\frac{R^{2}}{D_{⊥}\tau }\right){\left(\Delta -\frac{1}{3}\delta \right)}^{-1},$$

where *R* is the radius of the fibre, τ is half the echo time, and D_⊥_ is the intrinsic diffusivity in the direction perpendicular to the fibres.

## Supporting Information

S1 Fig Transform parameters from the motion and eddy-current correction versus volume number. Data were averaged across all subjects, for the conventional correction using FSL (A, red) and the CHARMED-based extrapolation-based procedure (B, blue). See Fig 5 for a corresponding plot and caption, for the two other correction methods.
(EPS)

S2 Fig Coronal sections. Column A: Non diffusion-weighted volume. Column B: Data corrected using references extrapolated using the CHARMED-model. Column C: Data corrected using references extrapolated using the CSF-corrected approach. The rows show a full coronal section (top), references (middle), and corrected data (bottom). The tissue outline from the non diffusion-weighted section is shown in yellow on top of the two extrapolated references and corrected data. Both the reference and the corrected data are displaced in the superior direction for the CHARMED-based approach (B) but not for the CSF-corrected approach (C).
(TIF)

## References

[1] Sundgren PC, Dong Q, Gómez-Hassan D, Mukherji SK, Maly P, Welsh R. Diffusion tensor imaging of the brain: review of clinical applications. Neuroradiology. 2004;46: 339–350. 10.1007/s00234-003-1114-x 15103435

[2] Assaf Y, Pasternak O. Diffusion tensor imaging (DTI)-based white matter mapping in brain research: a review. J Mol Neurosci. 2008;34: 51–61. 10.1007/s12031-007-0029-0 18157658

[3] Jensen JH, Helpern JA, Ramani A, Lu H, Kaczynski K. Diffusional kurtosis imaging: the quantification of non-gaussian water diffusion by means of magnetic resonance imaging. Magn Reson Med. 2005;53: 1432–1440. 10.1002/mrm.20508 15906300

[4] Westin CF, Szczepankiewicz F, Pasternak O, Özarslan E, Topgaard D, Knutsson H, et al Measurement Tensors in Diffusion MRI: Generalizing the Concept of Diffusion Encoding. Med Image Comput Comput Assist Interv. 2014;8675: 209–216. 10.1007/978-3-319-10443-0_27 PMC4386881 25320801

[5] Tournier JD, Calamante F, Gadian DG, Connelly A. Direct estimation of the fiber orientation density function from diffusion-weighted MRI data using spherical deconvolution. NeuroImage. 2004;23: 1176–1185. 10.1016/j.neuroimage.2004.07.037 15528117

[6] Tournier JD, Calamante F, Connelly A. Determination of the appropriate b value and number of gradient directions for high-angular-resolution diffusion-weighted imaging. NMR Biomed. 2013;26: 1775–1786. 10.1002/nbm.3017 24038308

[7] Nilsson M, van Westen D, Ståhlberg F, Sundgren PC, Lätt J. The role of tissue microstructure and water exchange in biophysical modelling of diffusion in white matter. Magn Reson Mater Phy. 2013;26: 345–370. 10.1007/s10334-013-0371-x PMC3728433 23443883

[8] Jensen JH, Falangola MF, Hu C, Tabesh A, Rapalino O, Lo C, et al Preliminary observations of increased diffusional kurtosis in human brain following recent cerebral infarction. NMR Biomed. 2011;24: 452–457. 10.1002/nbm.1610 20960579 PMC3549661

[9] Van Cauter S, Veraart J, Sijbers J, Peeters RR, Himmelreich U, De Keyzer F, et al Gliomas: diffusion kurtosis MR imaging in grading. Radiology. 2012;263: 492–501. 10.1148/radiol.12110927 22403168

[10] Haselgrove JC, Moore JR. Correction for distortion of echo-planar images used to calculate the apparent diffusion coefficient. Magn Reson Med. 1996;36: 960–964. 8946363 10.1002/mrm.1910360620

[11] Rohde GK, Barnett AS, Basser PJ, Marenco S, Pierpaoli C. Comprehensive approach for correction of motion and distortion in diffusion-weighted MRI. Magn Reson Med. 2004;51: 103–114. 10.1002/mrm.10677 14705050

[12] Leemans A, Jones DK. The B-matrix must be rotated when correcting for subject motion in DTI data. Magn Reson Med. 2009;61: 1336–1349. 10.1002/mrm.21890 19319973

[13] Andersson JLR, Skare S. A model-based method for retrospective correction of geometric distortions in diffusion-weighted EPI. NeuroImage. 2002;16: 177–199. 10.1006/nimg.2001.1039 11969328

[14] Nielsen JF, Ghugre NR, Panigrahy A. Affine and polynomial mutual information coregistration for artifact elimination in diffusion tensor imaging of newborns. Magn Reson Imaging. 2004;22: 1319–1323. 10.1016/j.mri.2004.08.024 15607105

[15] Maes F, Collignon A, Vandermeulen D, Marchal G, Suetens P. Multimodality image registration by maximization of mutual information. IEEE Trans Med Imaging. IEEE; 1997;16: 187–198. 9101328 10.1109/42.563664

[16] Ben-Amitay S, Jones DK, Assaf Y. Motion correction and registration of high b-value diffusion weighted images. Magn Reson Med. 2012;67: 1694–1702. 10.1002/mrm.23186 22183784

[17] Pasternak O, Sochen N, Gur Y, Intrator N, Assaf Y. Free water elimination and mapping from diffusion MRI. Magn Reson Med. 2009;62: 717–730. 10.1002/mrm.22055 19623619

[18] Poot DHJ, Dekker den AJ, Achten E, Verhoye M, Sijbers J. Optimal experimental design for diffusion kurtosis imaging. IEEE Trans Med Imaging. 2010;29: 819–829. 10.1109/TMI.2009.2037915 20199917

[19] Emre M, Aarsland D, Brown R, Burn DJ, Duyckaerts C, Mizuno Y, et al Clinical diagnostic criteria for dementia associated with Parkinson's disease. Mov Disord. 2007;22: 1689–1707. 10.1002/mds.21507 17542011

[20] Klein S, Staring M, Murphy K, Viergever MA, Pluim JPW. elastix: a toolbox for intensity-based medical image registration. IEEE Trans Med Imaging. 2010;29: 196–205. 10.1109/TMI.2009.2035616 19923044

[21] Bai Y, Alexander DC. Model-based registration to correct for motion between acquisitions in diffusion MR imaging. Biomedical Imaging: From Nano to Macro. 2008 10.1109/ISBI.2008.4541154

[22] Metzler-Baddeley C, O'Sullivan MJ, Bells S, Pasternak O, Jones DK. How and how not to correct for CSF-contamination in diffusion MRI. NeuroImage. 2012;59: 1394–1403. 10.1016/j.neuroimage.2011.08.043 21924365

[23] Mohammadi S, Tabelow K, Ruthotto L, Feiweier T, Polzehl J, Weiskopf N. High-resolution diffusion kurtosis imaging at 3T enabled by advanced post-processing. Front Neurosci. 2014;8: 427 10.3389/fnins.2014.00427 25620906 PMC4285740

[24] Bastin ME. On the use of the FLAIR technique to improve the correction of eddy current induced artefacts in MR diffusion tensor imaging. Magn Reson Imaging. 2001;19: 937–950. 11595365 10.1016/s0730-725x(01)00427-1

[25] Zhang H, Schneider T, Wheeler-Kingshott CA, Alexander DC. NODDI: practical in vivo neurite orientation dispersion and density imaging of the human brain. NeuroImage. 2012;61: 1000–1016. 10.1016/j.neuroimage.2012.03.072 22484410

[26] Lebel C, Gee M, Camicioli R, Wieler M, Martin W, Beaulieu C. Diffusion tensor imaging of white matter tract evolution over the lifespan. NeuroImage. 2012;60: 340–352. 10.1016/j.neuroimage.2011.11.094 22178809

[27] Lätt J, Nilsson M, Wirestam R, Ståhlberg F, Karlsson N, Johansson M, et al Regional values of diffusional kurtosis estimates in the healthy brain. J Magn Reson Imaging. 2013;37: 610–618. 10.1002/jmri.23857 23055442 PMC3596978

[28] Bennett KM, Schmainda KM, Bennett RT, Rowe DB, Lu H, Hyde JS. Characterization of continuously distributed cortical water diffusion rates with a stretched-exponential model. Magn Reson Med. 2003;50: 727–734. 10.1002/mrm.10581 14523958

[29] Szczepankiewicz F, Lasič S, van Westen D, Sundgren PC, Englund E, Westin C-F, et al Quantification of microscopic diffusion anisotropy disentangles effects of orientation dispersion from microstructure: applications in healthy volunteers and in brain tumors. NeuroImage. 2015;104: 241–252. 10.1016/j.neuroimage.2014.09.057 25284306 PMC4252798

[30] Mohammadi S, Möller HE, Kugel H, Müller DK, Deppe M. Correcting eddy current and motion effects by affine whole-brain registrations: evaluation of three-dimensional distortions and comparison with slicewise correction. Magn Reson Med. 2010;64: 1047–1056. 10.1002/mrm.22501 20574966

[31] Hansen B, Lund TE, Sangill R, Jespersen SN. Experimentally and computationally fast method for estimation of a mean kurtosis. Magn Reson Med. 2013;69: 1754–1760. 10.1002/mrm.24743 23589312

[32] Lätt J, Nilsson M, Brockstedt S, Wirestam R, Ståhlberg F. Bias free estimates of the diffusional kurtosis in two minutes: Avoid solving the kurtosis tensor. Proc Intl Soc Mag Reson Med. 2010;: 3972.

[33] Kamagata K, Tomiyama H, Motoi Y, Kano M, Abe O, Ito K, et al Diffusional kurtosis imaging of cingulate fibers in Parkinson disease: Comparison with conventional diffusion tensor imaging. Magn Reson Imaging. 2013;31: 1501–1506. 10.1016/j.mri.2013.06.009 23895870

[34] Tournier JD, Calamante F, Connelly A. MRtrix: Diffusion tractography in crossing fiber regions. Lee J, editor. Int J Imaging Syst Technol. 2012;22: 53–66. 10.1002/ima.22005

[35] Tournier JD, Yeh C-H, Calamante F, Cho K-H, Connelly A, Lin C-P. Resolving crossing fibres using constrained spherical deconvolution: validation using diffusion-weighted imaging phantom data. NeuroImage. 2008;42: 617–625. 10.1016/j.neuroimage.2008.05.002 18583153

[36] Jones DK, Christiansen KF, Chapman RJ, Aggleton JP. Neuropsychologia. Neuropsychologia. Elsevier; 2013;51: 67–78. 10.1016/j.neuropsychologia.2012.11.018 23178227 PMC3611599

[37] Andersson JL, Jenkinson M, Smith S. Non-linear registration, aka Spatial normalisation. FMRIB Analysis Group of the University of Oxford. FMRIB technical report; 2007. Report No.: TR07JA2.

[38] Liptrot MG, Sidaros K, Dyrby TB. Addressing the path-length-dependency confound in white matter tract segmentation. PLoS One. 2014;9: e96247 10.1371/journal.pone.0096247 24797510 PMC4010423

[39] Assaf Y, Basser PJ. Composite hindered and restricted model of diffusion (CHARMED) MR imaging of the human brain. NeuroImage. 2005;27: 48–58. 10.1016/j.neuroimage.2005.03.042 15979342

[40] Nam H, Park H-J. Distortion correction of high b-valued and high angular resolution diffusion images using iterative simulated images. NeuroImage. 2011;57: 968–978. 10.1016/j.neuroimage.2011.05.018 21600994

[41] Nilsson M, Lätt J, Nordh E, Wirestam R, Ståhlberg F, Brockstedt S. On the effects of a varied diffusion time in vivo: is the diffusion in white matter restricted? Magn Reson Imaging. 2009;27: 176–187. 10.1016/j.mri.2008.06.003 18657924

[42] Irfanoglu MO, Modi P, Nayak A, Hutchinson EB, Sarlls J, Pierpaoli C. DR-BUDDI (Diffeomorphic Registration for Blip-Up blip-Down Diffusion Imaging) method for correcting echo planar imaging distortions. NeuroImage. 2015;106: 284–299. 10.1016/j.neuroimage.2014.11.042 25433212 PMC4286283

[43] Sotiropoulos SN, Jbabdi S, Xu J, Andersson JL, Moeller S, Auerbach EJ, et al Advances in diffusion MRI acquisition and processing in the Human Connectome Project. NeuroImage. 2013;80: 125–143. 10.1016/j.neuroimage.2013.05.057 23702418 PMC3720790

[44] Crum WR, Griffin LD, Hill D, Hawkes DJ. Zen and the art of medical image registration: correspondence, homology, and quality. NeuroImage. Elsevier; 2003;20: 1425–1437. 10.1016/j.neuroimage.2003.07.014 14642457

[45] Szczepankiewicz F, Lätt J, Wirestam R, Leemans A, Sundgren PC, van Westen D, et al Variability in diffusion kurtosis imaging: impact on study design, statistical power and interpretation. NeuroImage. 2013;76: 145–154. 10.1016/j.neuroimage.2013.02.078 23507377

[46] Nilsson M, Lätt J, van Westen D, Brockstedt S, Lasič S, Ståhlberg F, et al Noninvasive mapping of water diffusional exchange in the human brain using filter-exchange imaging. Magn Reson Med. 2013;69: 1573–1581. 10.1002/mrm.24395 22837019

[47] Burton EJ, McKeith IG, Burn DJ, Williams ED, O'Brien JT. Cerebral atrophy in Parkinson's disease with and without dementia: a comparison with Alzheimer's disease, dementia with Lewy bodies and controls. Brain. 2004;127: 791–800. 10.1093/brain/awh088 14749292

[48] Chan L-L, Rumpel H, Yap K, Lee E, Loo H-V, Ho G-L, et al Case control study of diffusion tensor imaging in Parkinson's disease. J Neurol Neurosurg Psychiatry. 2007;78: 1383–1386. 10.1136/jnnp.2007.121525 17615165 PMC2095589

[49] Gattellaro G, Minati L, Grisoli M, Mariani C, Carella F, Osio M, et al White matter involvement in idiopathic Parkinson disease: a diffusion tensor imaging study. AJNR Am J Neuroradiol. 2009;30: 1222–1226. 10.3174/ajnr.A1556 19342541 PMC7051338

[50] Yoshikawa K, Nakata Y, Yamada K, Nakagawa M. Early pathological changes in the parkinsonian brain demonstrated by diffusion tensor MRI. J Neurol Neurosurg Psychiatry. BMJ Publishing Group Ltd; 2004;75: 481–484. 10.1136/jnnp.2003.021873 14966170 PMC1738942

[51] Zhan W, Kang GA, Glass GA, Zhang Y, Shirley C, Millin R, et al Regional alterations of brain microstructure in Parkinson's disease using diffusion tensor imaging. Mov Disord. 2012;27: 90–97. 10.1002/mds.23917 21850668 PMC4472452

[52] Theilmann RJ, Reed JD, Song DD, Huang MX, Lee RR, Litvan I, et al White-matter changes correlate with cognitive functioning in Parkinson's disease. Front Neurol. 2013;4: 37 10.3389/fneur.2013.00037 23630517 PMC3624087

[53] Compta Y, Parkkinen L, O'Sullivan SS, Vandrovcova J, Holton JL, Collins C, et al Lewy- and Alzheimer-type pathologies in Parkinson's disease dementia: which is more important? Brain. 2011;134: 1493–1505. 10.1093/brain/awr031 21596773 PMC4194668

[54] Child ND, Benarroch EE. Anterior nucleus of the thalamus: functional organization and clinical implications. Neurology. 2013;81: 1869–1876. 10.1212/01.wnl.0000436078.95856.56 24142476

[55] Douaud G, Jbabdi S, Behrens TEJ, Menke RA, Gass A, Monsch AU, et al DTI measures in crossing-fibre areas: increased diffusion anisotropy reveals early white matter alteration in MCI and mild Alzheimer's disease. NeuroImage. 2011;55: 880–890. 10.1016/j.neuroimage.2010.12.008 21182970 PMC7116583

[56] Bracht T, Jones DK, Müller TJ, Wiest R, Walther S. Limbic white matter microstructure plasticity reflects recovery from depression. J Affect Disord. 2014;170C: 143–149. 10.1016/j.jad.2014.08.031 25240841

[57] Lasič S, Szczepankiewicz F, Eriksson S, Nilsson M, Topgaard D. Microanisotropy imaging: quantification of microscopic diffusion anisotropy and orientational order parameter by diffusion MRI with magic-angle spinning of the q-vector. Frontiers in Physics. 2014;: 1–35.

[58] Yendiki A, Koldewyn K, Kakunoori S, Kanwisher N, Fischl B. Spurious group differences due to head motion in a diffusion MRI study. NeuroImage. 2013;88C: 79–90. 10.1016/j.neuroimage.2013.11.027 24269273 PMC4029882

[59] Assaf Y, Freidlin RZ, Rohde GK, Basser PJ. New modeling and experimental framework to characterize hindered and restricted water diffusion in brain white matter. Magn Reson Med. 2004;52: 965–978. 10.1002/mrm.20274 15508168

