# Supplementary figures and images for: Extrapolation-Based References Improve Motion and Eddy-Current Correction of High B-Value DWI Data: Application in Parkinson’s Disease Dementia
Source: PLoS One. 2015 Nov 3;10(11):e0141825. doi: 10.1371/journal.pone.0141825 (PMC4631453; doi:10.1371/journal.pone.0141825)

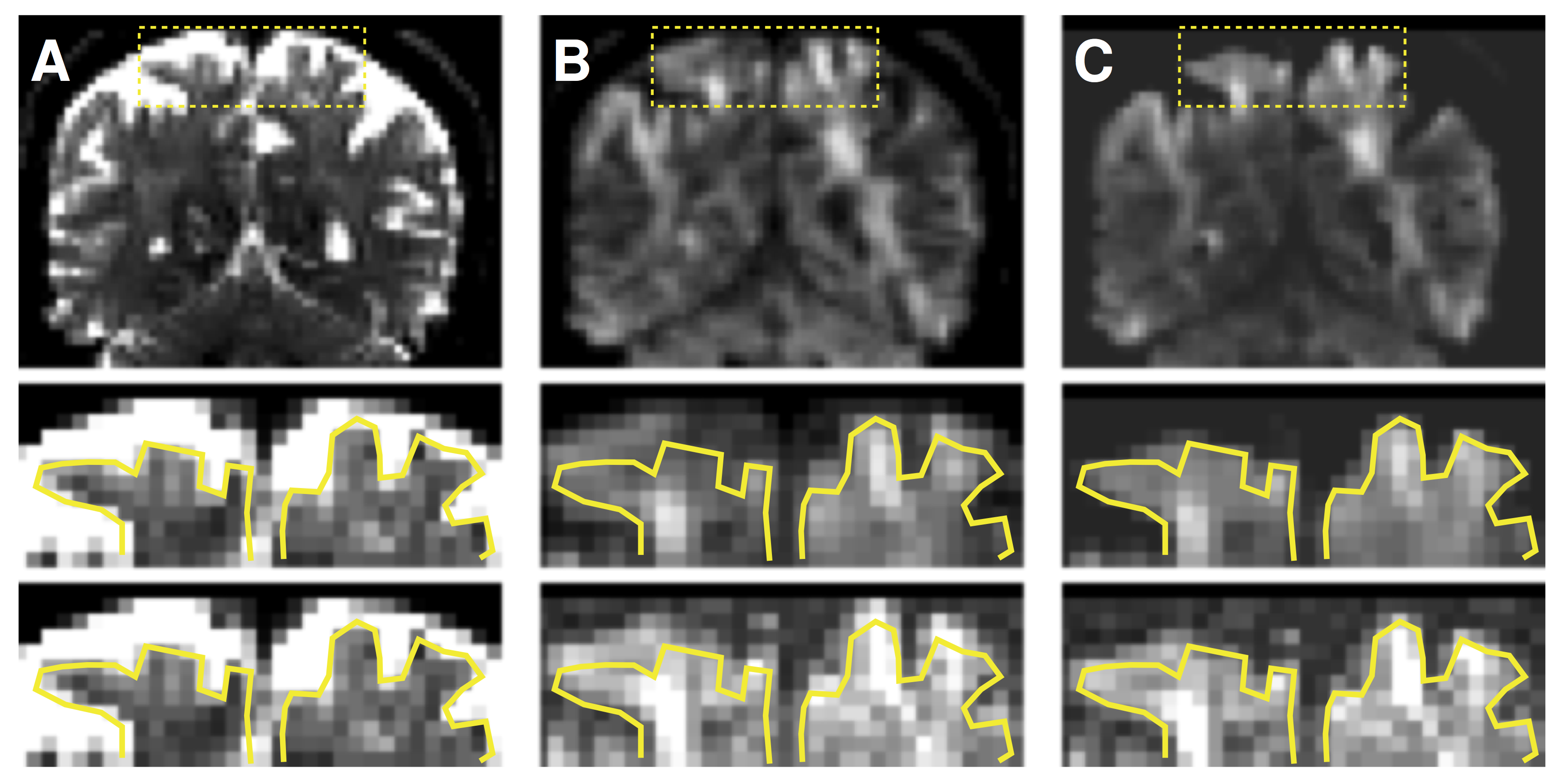

Supplement: S2 Fig — Column A: Non diffusion-weighted volume. Column B: Data corrected using references extrapolated using the CHARMED-model. Column C: Data corrected using references extrapolated using the CSF-corrected approach. The rows show a full coronal section (top), references (middle), and corrected data (bottom). The tissue outline from the non diffusion-weighted section is shown in yellow on top of the two extrapolated references and corrected data. Both the reference and the corrected data are displaced in the superior direction for the CHARMED-based approach (B) but not for the CSF-corrected approach (C). (TIF) [file pone.0141825.s002.tif]
